# Impact of a behaviour change intervention on long-lasting insecticidal net care and repair behaviour and net condition in Nasarawa State, Nigeria

**DOI:** 10.1186/s12936-014-0538-6

**Published:** 2015-01-21

**Authors:** Hannah Koenker, Albert Kilian, Gabrielle Hunter, Angela Acosta, Leah Scandurra, Babafunke Fagbemi, Emmanuel O Onyefunafoa, Megan Fotheringham, Matthew Lynch

**Affiliations:** Johns Hopkins Bloomberg School of Public Health Center for Communication Programs, Baltimore, MD USA; Tropical Health LLP, Montagut, Spain; Malaria Consortium, Montagut, Spain; Center for Communication Programs Nigeria, Abuja, Nigeria; Malaria Consortium, Abuja, Nigeria; US Agency for International Development, President’s Malaria Initiative, Washington, DC USA

**Keywords:** Malaria, Behaviour change communication, Care and repair, LLIN, ITN, Durability

## Abstract

**Background:**

While some data on net durability have been accumulating in recent years, including formative qualitative research on attitudes towards net care and repair, no data are available on how the durability of a net is influenced by behaviour of net maintenance, care and repair, and whether behavioural change interventions (BCC) could substantially impact on the average useful life of the net.

**Methods:**

The study used an intervention-control design with before-after assessment through repeated cross-sectional household surveys with two-stage cluster sampling following Nasarawa State’s December 2010 mass campaign. All campaign nets were 100-denier polyester, long-lasting insecticidal nets (LLIN). Baseline, midline, and endline surveys occurred at one-year intervals, in March 2012, March 2013, and April 2014, respectively. Outcome measures were the proportion of confirmed campaign nets with observed repairs, and the proportion in serviceable condition, measured with proportionate hole index (pHI) and according to WHO guidelines.

**Results:**

For all respondents, exposure to BCC messages was strongly correlated with increased positive attitude towards care and repair, and increases in attitude were positively correlated with observed net repairs, and with the proportion of nets in serviceable condition. In a multivariate regression model, positive care and repair attitude (OR 6.17 p = 0.001) and level of exposure (1 source: OR 4.00 p = 0.000; 3 sources: OR 9.34 p = 0.000) remained the most significant predictors of net condition, controlling for background and environmental factors. Nets that were tied up had 2.70 higher odds of being in serviceable condition (p = 0.001), while repairs made to nets were not sufficient to improve their pHI category. Estimated median net lifespan was approximately one full year longer for nets in households with a positive compared to a negative attitude.

**Conclusion:**

Exposure to multiple channels of a comprehensive BCC intervention was associated with improved attitude scores, and with improved net condition at endline. It is possible for BCC interventions to change both attitudes and behaviours, and to have an important effect on overall median net lifespan. Care and repair messages are easily incorporated into existing malaria BCC platforms, and will help contribute to improved net condition, providing, in principle, more protection from malaria.

## Background

Malaria prevention with long-lasting insecticidal mosquito nets (LLINs) has seen an intensive scale-up in sub-Saharan Africa in recent years. As many countries have now achieved high ownership coverage with LLIN and are approaching the universal coverage target of one net for every two people of the population at risk as recommended by WHO, the question of how these successes can be sustained, i.e., high coverage levels be maintained, becomes the focus of discussion. In this context the importance of net durability and the ‘average useful life’ of a net is increasingly recognized as one of the critical factors that determines the frequency at which nets need to be replaced. This is reflected in recent WHO guidelines for the monitoring of LLINs in the field, which outlines the issues and suggests methods of net assessment [1,2].

While data on net durability has been accumulating in recent years [3-9], including formative qualitative research on attitudes towards net care and repair [10-12], there is some indication that it may vary by environmental or climatic conditions. Allan *et al.* [13] found the physical condition of polyester nets in eastern Chad to be much poorer than would have been expected from similar nets seen in the more moderate climate of western Uganda [14]. While a 2006 case study describes a behavioural change communication (BCC) intervention and its effect on prevalence of repairs to nets [15], no data at all are available on how the durability of a net is influenced by behaviour of net maintenance, care and repair, and whether BCC could significantly impact on median net lifespan.

Improvements in knitting pattern or other aspects of textiles themselves have potential for extending net life [16], but have been difficult to implement due to lack of evidence for their impact on overall net life in field conditions, and procurement practices that maintain a focus on lowest price. Should improvements in net durability be possible via BCC interventions, the time period between net replacement could in theory be extended, leading to overall cost savings for program planners and donor agencies. At minimum, improved condition of nets would protect more people for longer periods of time between resupply.

## Methods

### Survey design

**Figure 1:**
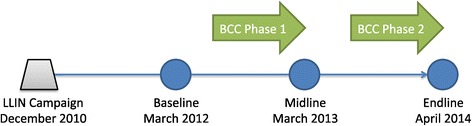
**Study timeline.**

The study used an intervention-control design with before-after assessment through repeated cross-sectional household surveys with two-stage cluster sampling following Nasarawa State’s December 2010 mass campaign, which aimed to deliver two LLINs per household. All campaign nets were 100-denier polyester. Baseline, midline (whose results are not reported here) and endline surveys occurred at one-year intervals, in March 2012, March 2013, and April 2014, respectively (Figure 1). The study was nested within a larger LLIN durability study in three states in Nigeria. Kokona Local Government Area (LGA) was selected as the intervention arm, with BCC activities as described below. Toto LGA, similar in environment and cultural aspects but out of the reach of the Nasarawa Broadcasting Service, was selected as the control site. Durability data and exposure to BCC messaging were collected from households in both sites.

For baseline and midline, a sample of 20 clusters with 15 households each (300 households) per site and time point was selected, using probability proportionate to size (PPS). Based on the interim results for the care and repair component the sample was increased for the control group (Toto LGA) for the endline survey to 28 clusters with 15 households each (420 households) in order to compensate for anticipated contamination for radio messaging in the control group, due to a planned extension of radio signal by the Nasarawa Broadcasting Service. The additional eight clusters were selected using PPS, after excluding the existing 20 clusters from the sampling frame. The targeted sample for the third round was 770 households bringing the targeted sample for the entire study to 2,170.

Clusters were selected once and maintained for all survey rounds, with an exception in the midline when six wards in the intervention LGA had to be replaced due to communal violence. Residents of these wards had either fled the area at the time of the midline or violence was still ongoing. For the endline, the six replacement wards added at midline were maintained. Households were newly selected in each cluster at each survey to minimize the Hawthorne effect of repeatedly interviewing the same households about net care and repair practices.

Sample size was calculated using an alpha error of 95%, a beta error of 80%, a design effect of 1.75, an anticipated non-response rate of 5%, and the expectation that households would own an average of 1.8 campaign nets at baseline, 1.5 at midline, and 1.0 at endline. These estimates were based on previous post-campaign surveys in Nigeria and on an assumed three-year average net survival.

### Study population

Respondents were adult members of the household, 18 years of age or older, usually the head of household or their spouse. Households had to have received at least one net from the December 2010 campaign to be eligible for interview.

### Procedures

A team of 20 interviewers were trained during a one-week training prior to each survey. Interviewers were retained by and large through each survey to provide consistency in data collection. Interviewers practiced translating the English questionnaire into Hausa to ensure all interviewers used consistent terminology, and training also included role-play, net hole assessment practice in the classroom and in local volunteer households, and pilot interviews.

Local authorities were informed as soon as clusters were selected and assisted in preparing communities for the interviews and in planning logistics. Fieldwork teams enumerated all households in the community, except in those larger than 200 households, where an equal-size section approach was used to first subdivide the community into neighbourhoods, and then selecting one of the sections for enumeration using a random-number list [17]. Households were then selected for interview using a random-number sheet, and the head of household interviewed if the household was eligible for participation. Three attempts were made to reach respondents and if the third was unsuccessful the household was replaced.

A structured questionnaire was used to gather data on household characteristics, nets received from the December 2010 campaign and any nets lost, net care and repair behaviour and attitudes, exposure to care and repair messages, and assessment of existing campaign nets. A visual aid for identification of LLIN brands was used, along with visual aids and plasticized tally sheets for the assessment of holes [18] according to WHO Guidelines for Monitoring the Durability off LLINs under Operational Conditions [1].

Holes were categorized as size one (0.5-2 cm in diameter), size two (2–10 cm), size three (10–25 cm) and size four (larger than 25 cm) per WHO guidelines [1,2]. The presence and number of repairs were also counted on each net.

Data entry was done using EpiData 3.1 software with double entry of all records. Both data sets were then compared and any discrepant records were verified from the original questionnaires. The data set was transferred to Stata 12 Statistical software package [19] for further consistency checks and preparation for analysis.

### Ethical consideration

Ethical approval was obtained from the Johns Hopkins School of Public Health Institutional Review Board (IRB #4108) and from the National Health Research Ethics Committee, Federal Ministry of Health in Nigeria (NHREC/01/01/2007). Respondents were informed about the purpose of the study in the local language (primarily Hausa) using a written script and the interview proceeded when verbal consent was given. This consent script contained information on the objectives of the survey, the risks, benefits and freedom of the participation, as well as information on confidentiality plus respondent rights.

### Analysis

The proportionate hole index (pHI) for each net was calculated per WHO guidelines using the following formula: pHI = (# size 1 holes) + (# size 2 holes × 23) + (# size 3 holes × 196) + (# size 4 holes × 578). Based on their pHI, nets were then categorized into the following conditions (note that ‘good’ is also included in ‘serviceable’).

- Good: total hole surface area <0.01 m^2^ or pHI < 64
- Serviceable: total hole surface area < =0.1 m^2^ or pHI < =642
- Too Torn: total hole surface area > 0.1 m^2^ or pHI > 642

Care and repair attitude scores were based on responses to eight statements using a Likert scale, where 1 was ‘strongly disagree’ and 4 was ‘strongly agree’. These were recoded during analysis to have −2 be ‘strongly disagree’ and +2 be ‘strongly agree’. Two statements were negatively phrased, and therefore were inversely recoded to make a positive response +2. Attitude scores for each respondent were summed and divided by eight to calculate an overall attitude score. Scores were then categorized into three groups: equal or less than zero (negative attitude); 0.01-1 (positive attitude), and 1.01-2 (very positive attitude).

The wealth index was computed at the household level using principal component analysis (PCA). Variables for household amenities, assets, livestock, and other characteristics that are related to a household’s socio-economic status were used for the computation. All variables were dichotomized except those of animal ownership where the total number owned was used. The first component of the PCA was used as the wealth index. Households were then classified according to their index value into quintiles.

Nets were assessed for brand labels, colour, and shape. Nets reported received from the campaign were counted as confirmed campaign nets, along with any other nets that matched the brand and colour distributed during the campaign. The main outcome indicators were the proportion of confirmed campaign nets with any observed repairs and the proportion of nets in serviceable condition at endline. Statistical analysis used logistic regression modelling techniques to assess potential associations between background characteristics, exposure to messages, attitude scores, and LLIN condition. All analysis accounted for sampling weights and adjusted standard errors for correlated data at the cluster level using the survey family commands in Stata. Pearson Chi-square tests were used to test for difference among categorical variables. The multivariate model was constructed using backwards elimination and Wald tests for significant parameters.

Estimates of the proportion of nets surviving at endline were calculated according to the WHO guidelines [1,2], taking into account the number of nets received from the campaign, the number lost due to wear and tear, and the number given away. Nets given away were excluded from analysis, as their ultimate fate was unknown. In addition, survival rates were adjusted for recall bias of number of nets received using the net/person ratio from the first post-campaign survey as an inflating factor (AK, personal communication). The proportion of nets surviving at endline was plotted against hypothetical decay curves [1,2] to determine the difference in estimated median survival between groups of nets.

### Behaviour change communication intervention

**Figure 2:**
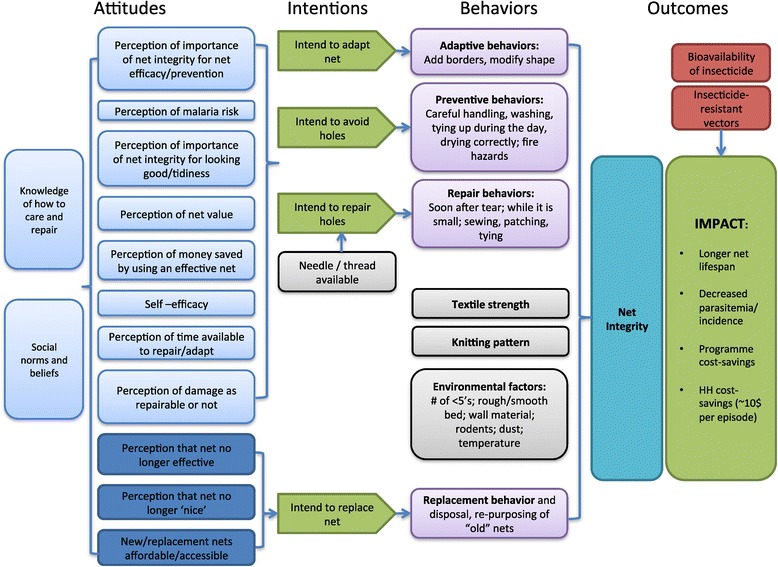
**Conceptual model for care and repair behaviours and outcomes.** Attitudes towards care and repair are shown on the left, leading to specific intentions and behaviours. Textile strength, knitting pattern, and environmental factors are outside the household’s control, but contribute to overall net integrity. Likewise, overall impact is also affected by bioavailability of insecticide on the net, and presence and proportion of insecticide-resistant vectors (not measured in this study).

The BCC intervention followed the P-Process©, a five-step planning process for behaviour change programs developed by JHUCCP [20]. The intervention used an evidence-based design, beginning with a conceptual model (Figure 2) used to design formative research and the overall intervention [12], and informed by existing research [15]. Through this process, the target audience, key behaviours, barriers, and motivators related to net care and repair were identified. These findings informed the BCC campaign strategy, which was developed by the Center for Communication Programs Nigeria, a Nigerian non-governmental organization. Materials and messages were designed at a participatory workshop with local malaria stakeholders, scriptwriters, and design experts. The target audience for the care and repair messaging were all adults who own and use mosquito nets, with a focus on women, as they were identified in the formative research as being primarily in charge of net care and repair duties [12]. The multi-channel BCC strategy consisted of advocacy, radio airings, interpersonal communication (IPC), and print materials. Radio spots, print materials, campaign logos, and key messages (Table 1) were pre-tested in focus groups in rural and urban communities and further refined before launch in the 20 focus communities of the intervention site.

**Table 1:** **Behaviour change intervention messages**

**Issue to address identified in formative research**	**Key message for BCC**
Specific sources of damage to nets	Fold or tie net away when not in use to keep out of reach of children, do not let children play with the net
	To avoid attracting rodents, do not soil a net with food, keep food away from nets
	Like a newborn baby, nets need to be handled with care
Lack of clarity on what are and how to carry out the concrete care and repair behaviours	You can tie, patch or stitch holes in nets
	Wash nets only when dirty and no more than once every three months, wash gently with mild soap
Making net care and repair a priority and incorporating into household routines	Nets are valuable and worth the time to care and repair
	A torn net can still be effective if repaired
	Repair small holes immediately
	When laying out the net for the evening, inspect it regularly for holes
	Holes in your net are like holes in your fence; if thieves (mosquitoes) can enter they may steal your health, money, sound sleep, and even your life

Campaign activities were conducted in two phases, from November 2012 to March 2013, and from December 2013 to March 2014. Findings of the midline assessment in March 2013 led to refinements in the campaign activities for the second phase.

### Radio

Radio content was broadcast exclusively on Nasarawa Broadcasting Service, (NBS), a local radio station selected because it reached the intervention site, but not the control site due to the strength of the station’s signal. However, during phase two of this BCC campaign, NBS upgraded its broadcasting signal, enabling its programming to reach into the control site.

The radio component focused on providing cues to action and reminder messages to reinforce the IPC activities. Five 60-second radio spots were produced, which were gradually introduced on the air during phase one. During phase two, only the three most popular spots were aired. In total, spots were aired over 800 times during the two phases. The airtime plan was initially prepared in coordination with the radio station, and subsequently refined based on a midline radio listenership assessment on peak listening times to the campaign radio station among the target audience. In phase two, 14 episodes of a 15-minute radio magazine show were produced and aired to reinforce the main messages, comprised of radio personality interviews with community leaders, community members, and recordings from community activities.

### Interpersonal communication (IPC)

For the IPC component, 40 community mobilizers were recruited and trained. Eligibility for recruitment consisted of the ability to read and write in English and living in or being very familiar with one of the 20 intervention communities. Community mobilizers attended a three-day training on malaria, care and repair behaviour, community mobilization skills, and on the conduct of campaign activities. A campaign manager or one of two campaign coordinators accompanied the mobilizers during most of their community activities for supportive supervision. Monthly meetings brought together all mobilizers and supervisors to review progress on the interpersonal activities, trouble-shoot challenges, ensure correct completion of field monitoring forms and reinforce concepts. Community mobilizers were paid a monthly stipend of 15,000 naira, which covered time and travel costs ($91).

IPC activities focused on modelling appropriate care and repair behaviours and consisted of house visits, community dialogues with street theatre, community outreach at weekly markets (‘market storms’), and road shows. During these events, community mobilizers shared the key messages, demonstrated net care and repair behaviour, and encouraged community members to stitch or patch demonstration nets. A song contest was organized in which all 20 communities composed and performed an original song about net care and repair in front of a live audience. A panel of judges selected the winning song, which was studio-recorded and broadcast over radio in phase two. The market storms and road shows were discontinued in phase two due to low effectiveness at reaching the target population during phase one, and IPC efforts (house visits and community dialogues) were intensified. In total, three community dialogues and an average of 87 house visits were conducted in each focus ward.

### Local advocacy

In the advocacy component, the campaign obtained the support of respected leaders, one of which participated in the recording of a radio spot encouraging families to care for and repair their nets. The chiefs of each of the 20 communities were approached during advocacy meetings to explain the BCC campaign and obtain their buy-in prior to conducting any community-level activities.

### Print

Finally, the print component consisted of five poster designs with photographs of community members modelling net care and repair behaviour, in particular demonstrating what a net looks like before, during and after repair. Other posters used photographs to demonstrate proper washing technique and ways to fold or tie a net out of harm’s way when not in use. These visual explanations were helpful to reach the low-literacy target audience. Posters were placed in public spaces in communities, in the nearest health facilities, and were used during public events. A printed job aid for the community mobilizers was also created, which listed frequently asked questions and answers regarding net care and repair, and served as a discussion guide for mobilizers during their IPC activities.

All of the above-referenced campaign materials are publically available in an online toolkit [18].

## Results

### Household characteristics

At baseline and at endline the two LGAs were similar in household size, number of children under five years old, household head level of education, radio and TV ownership, and ownership and population access of LLINs (Table 2). While the two LGAs were similar at baseline in the proportion of polygamous households, at endline the intervention area had 64.7% of households identifying as polygamous, against 42% of households in the control area. It is possible that this reflects the impact of communal violence in Nasarawa State, which occurred in the intervention area and affected implementation of some of the BCC activities (in both phases) in specific wards. Since households were selected based on having received at least one net during the December 2010 campaign, overall net ownership was high, increasing from 90 and 85% (for any net and any ITN, respectively, at baseline) to 100 and 97.5%, respectively, at endline, likely reflecting health facility, community LLIN distributions, and retail sales that were ongoing during the period. Population access to an LLIN (the proportion of people with access to an LLIN within their household) had decreased only slightly from baseline to endline, from 44.9 to 41.3% in the control area and from 41.1 to 40.7% in the intervention area.

**Table 2:** **Household characteristics for control and intervention sites at baseline and endline**

	**N**	**Baseline control (%)**	**Baseline intervention (%)**	p*	**n**	**Endline control (%)**	**Endline intervention (%)**	p*
No. of children under five	432			0.219	539			0.737
1		30.4	41.4			34.8	34.6	
2		36.4	32.1			30.4	30.0	
3		15.7	14.4			19.9	17.1	
4+		17.5	12.1			14.9	18.4	
Household is polygamous	591	57.6	47.6	0.291	709	42.0	64.7	0.0002
Level of education of head of household	591			0.465	709			0.345
Non-literate		52.2	49.0			52.7	47.5	
Some primary		14.5	18.7			22.1	27.3	
Some secondary		21.2	24.8			20.6	17.5	
Some higher education		12.1	7.5			4.6	7.7	
Own radio	591	74.4	83.0	0.071	709	87.4	85.5	0.669
Own TV	591	36.7	33.7	0.686	709	23.1	29.0	0.329
Own at least 1 mosquito net	591	92.9	87.8	0.304	709	100	100	1.000
Own at least 1 LLIN	591	87.5	82.6	0.409	709	98.1	97.0	0.442
Population access to an LLIN within the household	591	44.9	41.3	0.076	709	39.0	40.7	0.839
HH ever had hole in nets	576	49.5	45.3	0.576	709	76.5	72.7	0.555
From tears	273	37.1	40	0.6387	531	57.1	50.9	0.4342
From corner ripping	273	20.3	14.6	0.3082	531	42.9	40.7	0.6917
From burns	273	2.1	6.2	0.1048	531	11.8	8.3	0.2527
From rats	273	17.5	27.7	0.1766	531	60.6	55.6	0.439

*Pearson Chi-square.

Households reported an increase in ever having experienced holes in their nets, from roughly half of households at baseline to three-quarters of households at endline, with no differences between control and intervention areas. The majority of holes were due to tears (baseline and endline), and more holes were attributed to rat damage at endline (58.6% of all households reported this type of damage) than at baseline. Burns were not a major source of damage in this area.

### Intention to treat analysis

As shown in Table 3, the per cent of campaign nets with any observed repairs between control and intervention sites was statistically similar at endline (p = 0.128). From baseline to endline, the per cent of nets with observed repairs increased in both areas, from 10.3 to 17.8% in the control area (p = 0.11) and 10.5 to 27% at endline in the intervention area (p = 0.003). However, proportions of nets in good, serviceable, and torn condition were statistically the same in the two LGAs at endline. As expected, an overall deterioration in net condition was observed at endline, with nets in serviceable condition falling from 90% at baseline to just over 50% at endline. Given the similarity of net condition between study areas, an analysis by exposure was conducted to assess differences by exposure to the campaign.

**Table 3:** **Condition of confirmed 2010 campaign nets in 2012 baseline survey and 2014 endline survey**

**Net characteristics**	**Baseline control**	**Baseline intervention**	**p**	**Endline control**	**Endline intervention**	**p**
n	156	134		443	253	
Any observed repairs (among nets with holes)	10.3	10.5	0.961	17.8	26.5	0.128
n	425	376		522	327	
% of nets in good condition	79.1	82.7	0.443	30.7	33.3	0.684
% of nets in serviceable condition	92.5	92.0	0.862	51.5	55.7	0.514
% of nets too torn for use	7.5	8.0	0.862	48.5	44.3	0.514

P-values are for comparisons between control and intervention areas for each survey, using a Pearson Chi-square test.

### Analysis by exposure

#### Evidence of contamination

**Figure 3:**
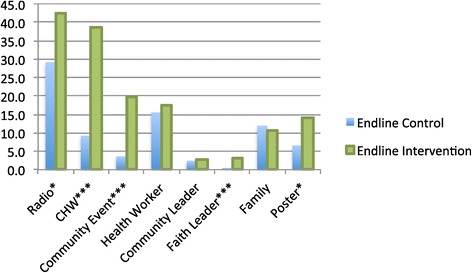
**Sources of messages in each study site (endline only).**

The BCC intervention was targeted only in Kokona LGA, where there was significant recall of care and repair messages, with 72.7% of respondents reporting hearing any care and repair message in the last six months. However, exposure to messages was also relatively high in the control LGA (Toto), at 46.8% (Table 4). Figure 3 shows the sources of messages in each LGA. As expected, radio, community health worker (CHW), community event and health workers were the main sources of exposure in the intervention area. In the control area, radio (29.1%) and health workers (15.5%) were the primary sources of exposure, indicating that indeed the boost in signal strength of NBS resulted in care and repair messages reaching the control LGA. Health workers in the control LGA were not trained in care and repair messages, but may have advised patients on this spontaneously, or possibly because they had been exposed to the campaign themselves. Community leaders and faith leaders were the least cited sources of care and repair information, and family members were a small but equally prevalent source in both areas.

**Table 4:** **Exposure to care and repair messages (endline)**

	**N**	**Baseline control**	**Baseline intervention**	**p**	**n**	**Endline control**	**Endline intervention**	**p**
Heard/saw any messages about care and repair	591	0.3	1.4	0.176	709	46.8	72.7	0.0005
Dose (No. of sources cited)	591				709			0.0001
0		99.7	98.6	0.176		53.2	27.3	
1						25.7	23.6	
2		--	--			12.6	28.0	
3+		--	--			8.5	21.2

**Figure 4:**
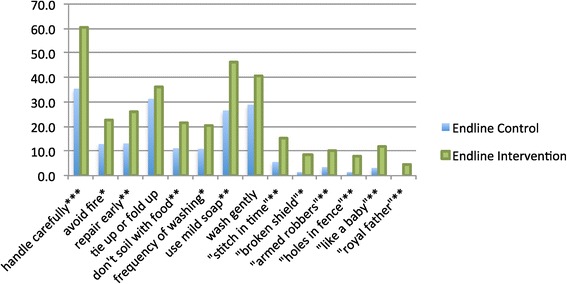
**Recall of messages on care and repair (endline only).**

Figure 4 shows the per cent of respondents who spontaneously recalled various messages used in the BCC intervention. Recall of messages was higher in the intervention than in the control area, but significant recall was observed in the control area, particularly for preventive messages including ‘handle carefully’, ‘tie up/fold up’ and messages on gentle washing. Patterns of recall were the same in both areas for specific messages. As is the norm in BCC campaigns, recall was lower for the specific radio spots and slogans than for the general key messages.

**Figure 5:**
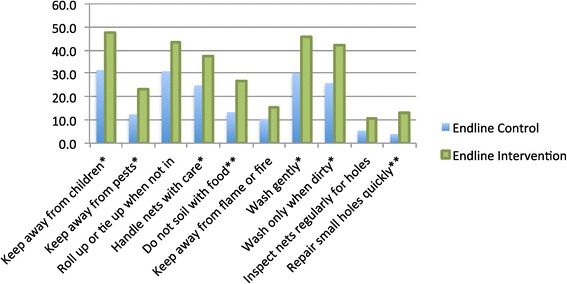
**What if anything do you do at home to prevent nets from tearing or getting holes?** **(endline only).**

Respondents were also asked what behaviour they do at home to take care of nets in the endline survey (Figure 5). The most common were ‘keep away from children’, ‘wash gently’, ‘roll up or tie up when not in use’, ‘wash only when dirty’ and ‘handle nets with care’. Significantly more respondents in the intervention area than the control area cited each behaviour. Likewise, significantly more respondents in the intervention area were able to list the recommended ways of washing a net, e.g. “gently” (p = 0.001), “in a basin” (0.002), and “with mild soap” (p = 0.005).

### Attitudes

A series of eight Likert-scale statements were presented to respondents, who then indicated whether they strongly disagreed, disagreed, agreed, or strongly agreed with the statement. The statements measured self-efficacy to repair holes and to do so immediately, confidence in repaired nets to remain effective, perceived social norms of net repair, and perception of nets as valuable. Attitude scores in the intervention area were significantly higher for confidence to repair holes immediately, to make the net last longer, and for perceived social norms (Table 5). Overall attitude scores were relatively high in each area, with 61 and 56% of respondents with positive attitudes in control and intervention, respectively, and 27 and 36% with very positive attitudes (Table 5).

**Table 5:** **Distribution of attitude scores by intervention area (endline only)**

**Grouped composite attitude scores**	**N**	**Control**	**Intervention**	**P=**
Positive attitude group	692			0.270
−2 to 0 (negative)		12.1	8.7	
0.01 to 1 (positive)		61.1	55.6	
1.01 to 2 (very positive)		26.9	35.7	
**Specific attitudes (range: −2 to 2)**	708			
Nets are valuable*		1.87	1.81	0.038
There are actions I can take to make my net last long**		1.21	1.37	0.018
*It is not possible to repair holes in nets*		−0.49	−0.44	0.595
A repaired net can still be effective against mosquitoes		0.93	1.09	0.055
Other people in this community fix holes in their mosquito nets***		−0.40	0.11	0.000
*I do not have time to repair a hole in my net*		−0.07	0.00	0.465
I can help protect my family from malaria by taking care of my net		1.47	1.51	0.501
I am confident I can repair holes immediately***		−0.02	0.48	0.000

Italics indicate negatively-phrased question; inverted when calculating composite attitude score. Pearson chi-square. *p<0.05, **p<0.01, ***p<0.001.

### Response bias

Self-reported repair within the last six months was compared to observed repairs on the nets themselves to check for response bias. At baseline, the rates of discontinuity were similar: around 4% of respondents reported repairs where none were observed, and 4% did not report repairs yet repairs were observed. At endline, rates of reported repair without observed repair rose to 11% overall, and were 14% in the intervention LGA *vs* 8% in the control LGA. Although it is possible that repairs were overlooked during observations, these results may indicate some response bias was present for self-reported repair, particularly in the intervention LGA. For this reason, only observed repairs and net condition (based on pHI) were included as outcome indicators, to be conservative.

### Analysis by exposure using the entire sample

Given the high levels of exposure in both intervention and control areas, outcome indicators were assessed by exposure to the campaign rather than by study area, to determine the overall effects of the BCC messages on attitudes and net condition.

### Exposure is correlated with positive attitude

**Figure 6:**
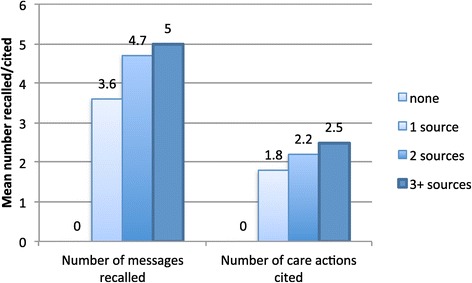
**Mean number of messages recalled and care actions cited, by number of sources mentioned.**

Dose–response relationships were assessed for exposure to the BCC campaign (number of sources of information cited) and for number of messages recalled, and with net care and repair attitude groups. The number of sources cited was closely associated with the number of messages recalled in Figure 6 (regression coefficient 1.67; p < 0.000) and with the number of net care actions the respondent cited (Figure 6).

**Figure 7:**
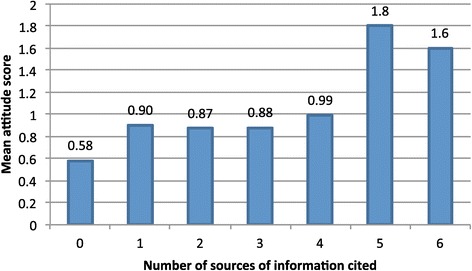
**Mean attitude score, by number of sources cited (endline).** Regression coefficient 0.13, p < 0.0001.

Attitude scores also increased with an increasing number of sources cited (Figure 7). These were linearly correlated with a regression coefficient of 0.13 (p < 0.000).

### Attitude is correlated with observed repair and net condition

**Figure 8:**
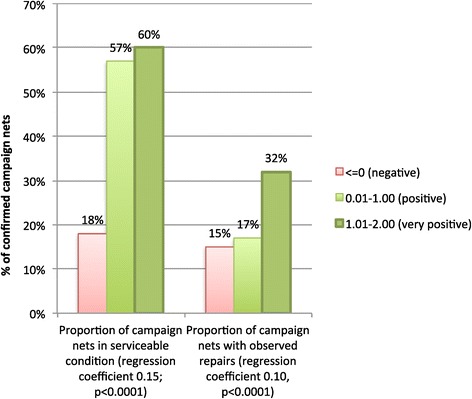
**Positive relationship between attitude scores and condition of nets (left) and observed repairs (right).**

Attitude scores were in turn correlated with increased observed repairs to campaign nets themselves, and with the proportion of campaign nets in serviceable condition. Only 18% of nets were in serviceable condition for those in the ‘negative attitude’ category, compared to 57% of nets for those with ‘positive attitude’, and 60% for ‘very positive’ attitude (Figure 8).

### Median estimated net lifespan

**Figure 9:**
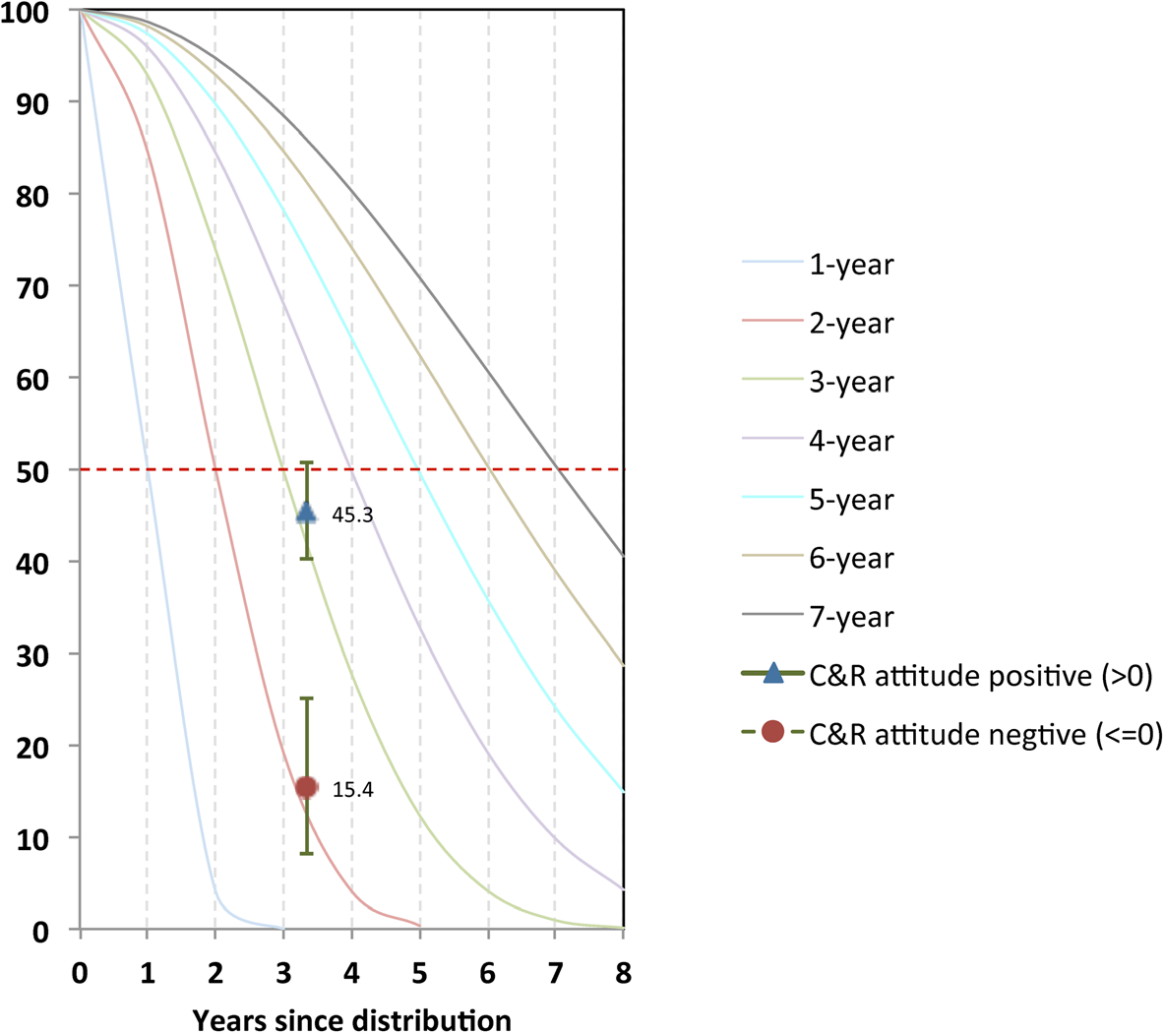
**Plot of proportion of campaign nets surviving at endline (3.3 years after distribution) by attitude score against standard decay curves.** Decay curves are labelled according to where each curve hits the median survival time (dotted red line at 50%), e.g. the green curve crosses the median at 3 years since distribution, and is therefore the curve for a 3-year net.

**Figure 10:**
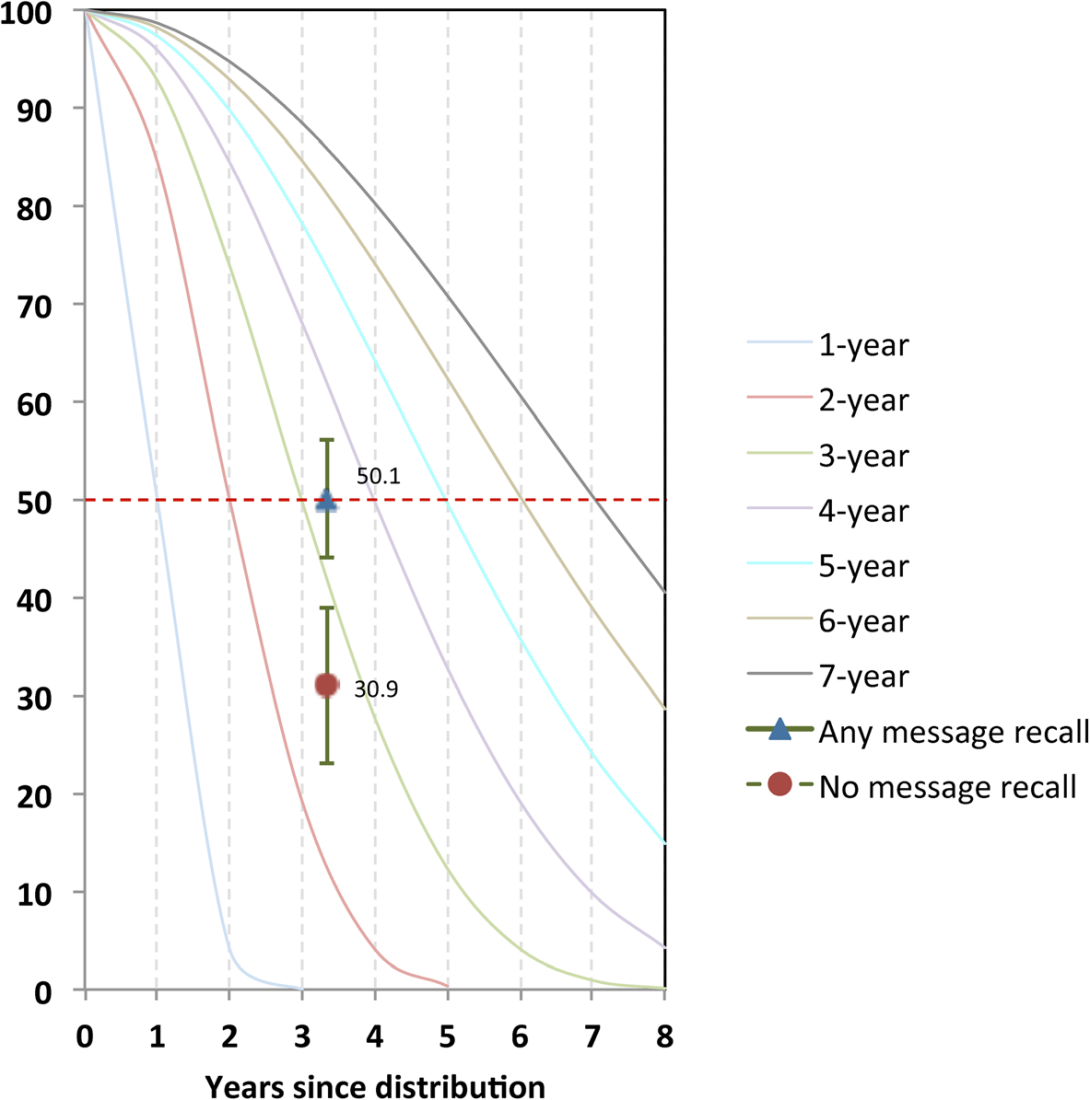
**Plot of proportion of nets surviving at endline (3.3 years after distribution) by exposure to the BCC intervention against standard decay curves.** Decay curves are labelled according to where each curve hits the 50% line (dotted red line), e.g. the green curve crosses the median at 3 years since distribution, and is therefore the curve for a 3-year net.

Three years after LLIN distribution, significantly higher proportions of nets survived among households with a net care and repair attitude score greater than zero (45.3%) as compared to households with attitude scores of zero or less (15.4%), shown in Table 6. Similarly, when attitude scores were further broken down into categories, a trend towards dose–response was observed, although this was not statistically significant (Table 6 middle section). When dichotomized by recall of messages, 30.9% of nets in households with no exposure to the care and repair campaign survived to endline, compared to 50.1% of nets for exposed households. Net survival was then plotted against hypothetical survival curves of defined median and median net lifespan estimated in households with positive and negative attitude (Figure 9). This indicated an approximately 1.0 year difference in median net lifespan for nets in households with positive attitudes, giving a two-year estimated median lifespan for nets in households with negative attitude toward care and repair, and a three-year estimated lifespan for those in households with a positive attitude. The estimated median lifespan difference was slightly less but still statistically significant for exposed *vs* unexposed households, where a difference of approximately 0.7 years was observed (Figure 10). This translates into a nine-month longer estimated lifespan for nets in households exposed to the campaign.

**Table 6:** **Estimated % of campaign nets in serviceable condition at endline, by attitude score and by level of exposure to BCC messages (both study sites)**

**Attitude score for care and repair**	**% of confirmed campaign nets in serviceable condition at endline**	**95% CI**
≤0	15.4%	8.1-5.0
0.01-1.0	45.4%	39.6-51.3
1.01-2.0	45.3%	39.7-50.9
0.01-2.0	45.3%	40.1-50.7
**Attitude score for care and repair**		
≤0	15.4%	8.1-25.0
0.01-0.74	40.3%	33.5-47.5
0.74-1.49	47.5%	41.0-54.1
1.5-2.0	51.5%	37.3-65.7
**Messages recalled**		
0	30.9%	23.1-39.0
≥1	50.1%	44.0-56.1

### Multivariate regression

Multivariate logistic regression was performed with the confirmed campaign nets at endline to determine relative contributions of attitudes and exposure to messages, controlling for background variables and predictors selected by using the conceptual model in Figure 2. Univariate analysis was first performed for these predictors, which included positive attitude, evidence of repair, whether the net was observed tied up, frequency of washing, presence of rodents in the house, type of sleeping place, exposure to the BCC campaign, poorest wealth quintile, education level of the head of household, gender of the respondent, polygamous household, drying location, and intervention area. Backwards elimination using Wald tests to assess significant predictors resulted in the multivariate model shown in Table 7. Head of household education, household being polygamous, frequency of washing, drying location, presence of rodents, and type of sleeping place were not significantly associated with net condition and were not included in the multivariate model.

**Table 7:** **Multivariate logistic regression for confirmed campaign nets in serviceable condition at endline in both study sites**

**Confirmed campaign net is in serviceable condition**	**Odds Ratio**	**95% CI**	**p**
Positive attitude towards care and repair	6.17	2.19-17.36	0.001
Net has any observed repair	0.36	0.18-0.73	0.005
Net is tied up	2.70	1.50-4.86	0.001
Dose			
1 source	4.00	2.30-6.94	0.000
2 sources	2.67	1.35-5.31	0.006
3+ sources	9.34	3.75-23.29	0.000
# of children under five	0.82	0.69-0.97	0.022
Poorest Quintile	0.47	0.24-0.95	0.035
Respondent is the spouse	1.62	1.00-2.62	0.05
Intervention LGA	0.48	0.24-0.97	0.04

Attitudes and level of exposure to the campaign were the strongest predictors of net condition (Table 7). Nets in households with positive attitude were 6.17 times as likely as those in households with negative attitude to be in serviceable condition. The number of sources recalled remained significant at one and three+, with 4.00 greater odds of nets being in serviceable condition for households reporting one source compared to no exposure, and 9.34 times greater odds for nets in households reporting three or more sources of information. Wealth quintile was also significant, with nets in the poorest quintile having half the odds of being serviceable compared to nets in the upper wealth quintiles. Nets that were hanging tied up had 2.70 greater odds of being in serviceable condition, while nets with observed repairs were in poor condition, with an odds ratio of 0.36. The number of children under five in the household was associated with a 0.82 reduction in odds of the net being serviceable. The respondent being the spouse resulted in 1.62 greater odds of the net being in serviceable condition, reflecting gender influence potentially related to exposure and attitudes.

In the same model, specific attitudes were assessed as predictors of nets in serviceable or better condition, shown in Table 8. General self-efficacy to repair, and response efficacy (confidence in a repaired net to protect against malaria) were both significant predictors of serviceable condition of nets. Self-efficacy to repair nets immediately and perceived social norm of net repair were not predictive. The perception that nets were valuable negatively predicted net condition, although this attitude was extremely positive for nearly all respondents, and may reflect the overall condition of nets in the sample.

**Table 8:** **Specific attitudes predicting serviceable condition of campaign nets, using the same regression model as Table** 7

**Confirmed campaign net is in serviceable condition**	**Odds ratio**	**95% CI**	**p**
**There are actions I can take to make nets last long	1.61	1.25-2.08	0.001
**A repaired net is still effective against mosquitoes	1.62	1.25-2.10	0.001
Others in this community repair holes in nets	0.85	0.64-1.13	0.255
*I do not have time to repair holes in my net*	0.92	0.78-1.09	0.314
I can help protect my family from malaria by taking care of my net	1.10	0.74-1.63	0.629
I am confident I can repair holes immediately	1.00	0.74-1.34	0.985
**Nets are valuable	0.38	0.21-0.69	0.002
*It is not possible to repair nets*	1.15	0.90-1.45	0.254

Italics indicate negatively-phrased question; inverted when calculating composite attitude score. *p<0.05, **p<0.01, ***p<0.001.

Further regression by type of exposure showed that nets in households with only radio exposure had 2.77 times greater odds of being in serviceable condition (p = 0.007), while only IPC exposure (either community health worker, community event, or health worker) was not significantly predictive (Table 9). The combination of radio and at least one IPC exposure was also significantly predictive, but with a lower odds ratio of 1.82 (p = 0.042). This likely reflects the skewed application of radio in the control LGA with absence of other channels.

**Table 9:** **Types of exposure predicting serviceable condition of campaign nets, using same regression model as Table** 7

**Confirmed campaign net is in serviceable condition**	**Odds ratio**	**95% CI**	**p**
Type of exposure (vs none)			
Radio only	2.77	1.34-5.74	0.007
IPC only	1.63	0.82-3.25	0.160
Radio and IPC	1.82	1.02-3.24	0.042
Other	1.80	0.77-4.16	0.168

## Discussion

For all respondents, exposure to the net care and repair BCC campaign was strongly correlated with increased positive attitude towards net care and repair, and increases in attitude were strongly correlated with self-reported actions taken to care for nets, with observed net repair, and with the proportion of nets in serviceable condition three years after net distribution. Estimated median net lifespan was approximately one full year longer for nets in households with a positive attitude compared to a negative attitude towards care and repair.

Intention to treat analysis indicated no difference between intervention and control areas in terms of observed repair or net condition. As other studies have found [21], it is often difficult to successfully implement experimental designs for BCC interventions due to leakage of messages into control areas. In this case, the boosting of the radio signal was factored into the design of the endline survey, and additional clusters were added to allow for the subsequent analysis by exposure.

At baseline, care and repair behaviours were quite rare, and the formative research also indicated that LLIN users in the study areas had not considered repair or care as a necessary activity [12].

Analysis by exposure found that indeed radio exposure was common (47%) in the control area, and that health workers were also a small but significant source of information in that area, indicating that the radio station was reaching into Toto. It is unclear whether health workers received other training including care and repair messaging or also heard the radio messages, or whether they spontaneously were recommending similar key messages. It is plausible that health workers may have heard the radio spots and been inclined to pass messages along to those receiving nets at health clinics.

Regression analysis showed that the number of channels through which respondents were exposed to BCC messaging, and their attitudes about care and repair were predictive of nets being in serviceable condition at the endline survey. Attitudes and level of exposure were the strongest predictors in the multivariate model. Given that exposure to radio was a better predictor of serviceable condition than IPC, it is possible that radio alone would be sufficient to promote care and repair behaviour. Radio has been shown to be a cost-effective way of changing behaviour, as it reaches many more people than community activities [22]. Nonetheless, the data also support the central tenet of BCC strategy design that the reinforcing properties of using multiple channels are more effective than using a single channel [23,24].

Other significant predictors in the regression model were the number of children under five in the household, poorest wealth quintile, and tying up the net during the day, echoing findings from the durability study within which the present study was nested (Kilian *et al.*, in prep). Children under five are skilled at breaking all kinds of household items; preventing their access to the net by tying it up during the day is likely a key strategy for prevention of tears. The data also indicate that preventive behaviour is likely to be more effective than repair behaviour in overall contribution to net longevity. Indeed, nets with observed repairs were in worse condition, most likely indicating that repairs occur at a late stage, once a net is already quite torn, and are not done at a level sufficient to improve the pHI category of the net from ‘torn’ to ‘serviceable’. The fact that the repair messages were disseminated when nets were already 2.5 years old is a likely contributor to this result; future studies should examine the role of BCC messaging on repair early in the net lifespan.

Frequency of washing and drying practices were not significant predictors of net condition, indicating that these behaviours may not be as damaging to nets as previously suspected, or that they are being conducted with care already. The type of sleeping place was significant in the univariate model but not in the final model, most likely because it is closely correlated with wealth quintile.

Care and repair behaviours are non-controversial and common sense, and therefore seem fairly easy to promote, particularly compared to other health behaviour such as condom use or uptake of family planning. The only other study on this topic found that a BCC intervention was able to increase the per cent of holes in nets that were repaired from 27% to 41% over six months in The Gambia, although repair did not have any effects on the numbers of mosquitoes caught at dawn under the nets [15]. Similar to that study, the present BCC intervention encouraged small do-able actions, mainly tying the net up during the day to keep away from young children and rodents, and treating it carefully. It is plausible that given the relative simplicity of the actions needed, that only a small ‘push’ from the BCC intervention was needed to encourage these behaviours. This is borne out by the results of the regression of serviceable condition on the different types of attitudes. Where self-efficacy and perception that repair took only a little time were strong, nets were more likely to be in serviceable condition at endline.

While this BCC intervention was conducted as a stand-alone intervention, its key components, the radio spots, CHW job aids and key messages, and community events, are relatively easily incorporated into existing malaria BCC platforms. Particularly for malaria prevention programmes that already train and support CHWs, the production of simple job aids is inexpensive, and key messages can be added into their routine work. Given the impact of radio-only exposure on serviceable condition of nets, planners may want to consider developing two to three short spots and adding them into the rotation of net use messaging, even if other community platforms are not available.

Formative research conducted in Senegal, Mali, Nigeria, and Uganda shows that attitudes around net care and repair are largely similar across these settings prior to BCC interventions [10-12,25,26]. Care and repair behaviours are not widespread and while respondents expressed anxiety about neighbours seeing their own torn or dirty nets, or view those with such nets as careless or lazy, there is also a clear lack of urgency to complete repairs. As attitudes, perceptions, and behaviours around net care and repair in these other settings were similar to Nigeria, it is reasonable that BCC interventions in other areas would potentially have similar impact, even where formative research reveals a lack of current care and repair practices.

While these results show that repairs made to nets did not improve their condition, this does not necessarily imply that BCC messages on repair should be dropped. Certainly the key message for program planners is that care and prevention help the most, but continuing to include some key messages on net repair is unlikely to hurt, and may contribute overall to a positive attitude towards care of the net, as evidenced by specific attitudes about repair remaining significantly predictive of net condition at endline.

Improved care practices are a ‘low-hanging fruit’ for LLIN longevity and malaria prevention. There are limited opportunities and technologies that allow LLINs to last longer in the field; apart from improvements in knitting pattern, procurement practices based primarily on lowest price per LLIN prevent manufacturers from investing in more expensive but more durable textiles. This study demonstrates that there are substantial gains to be made in median net lifespan simply by improving the way that nets are handled within households. While these results alone are unlikely to change current procurement timelines, improved net care can contribute to maintaining cohorts of nets in better overall condition within a given timeframe, leading to higher rates of net use between mass distributions, as one example, or prior to replacement via continuous distribution channels. Improved net condition should also provide better protection from malaria. Further research is urgently needed to assess the relationship between malaria parasitaemia, malaria cases, and the proportionate hole index of LLINs. This would help in being able to set more evidence-based thresholds for ‘good’, ‘serviceable’, and ‘too torn’ nets, and help relate net condition to public health impact.

## Conclusion

Exposure to multiple channels of a comprehensive BCC intervention was associated with improved net care and repair attitude scores, and with improved net condition at endline. It is possible for BCC interventions to change both attitudes and behaviour, and to have a significant effect on overall median net lifespan. Care and repair messages are easily incorporated into existing malaria BCC platforms, and will help contribute to improved net condition, providing, in principle, more protection from malaria.
